# Analysis of the Clinical Features of Intrauterine *Ureaplasma urealyticum* Infection in Preterm Infants: A Case-Control Study

**DOI:** 10.3389/fped.2021.774150

**Published:** 2021-12-09

**Authors:** Tong Sun, Jianhua Fu

**Affiliations:** Department of Pediatrics, Shengjing Hospital of China Medical University, Shenyang, China

**Keywords:** intrauterine infection, *Ureaplasma urealyticum*, preterm infants, bronchopulmonary dysplasia, retinopathy of prematurity, prematurity metabolic bone disease

## Abstract

**Objective:** To analyze the clinical characteristics of intrauterine *Ureaplasma urealyticum* (UU) infection in premature infants.

**Method:** In this single-center retrospective case-control study, 291 preterm infants born in our hospital and hospitalized in our department and gestational age no more than 32 weeks, birth weight no more than 2000 g were included from January 2019 to January 2021. Lower respiratory tract secretion, gastric fluid and urine were collected for UU RNA detection within 48 h after birth. Intrauterine UU infection is defined by at least one positive UU-PCR test of secreta or excreta of preterm infants after birth. The UU infection group included 86 preterm infants and the non-UU infection group included 205 preterm infants. We compared their clinical features, hemogram changes and disease outcomes using statistical analyses.

**Results:** The clinical characteristics of premature infants such as the duration of oxygen use and ventilator use in hospital were significantly prolonged in the UU infection group (*P* < 0.05). The levels of leukocytes, platelet and procalcitonin in the UU infection group were significantly higher than in the non-UU infection group (*P* < 0.05). In terms of preterm complications, only the incidences of bronchopulmonary dysplasia, retinopathy of prematurity and metabolic bone disease in premature infants in the UU infection group were significantly higher than those in the non-UU infection group (*P* < 0.05). The mode of delivery, maternal premature rupture of membranes, and postnatal leukocyte level were independent risk factors for UU infection, while gestational hypertension was a protective factor for UU infection. The level of leukocytes in postnatal hemogram of premature infants could be used as a diagnostic index of UU infection, but the diagnostic accuracy was poor.

**Conclusion:** In our study, UU infection can increase the incidence of bronchopulmonary dysplasia, retinopathy of prematurity and metabolic bone disease in preterm infants, but have no effect on the incidence of necrotizing enterocolitis, intracranial hemorrhage, white matter damage and other diseases in preterm infants. For high-risk premature infants, UU should be detected as soon as possible after birth, early intervention and drug treatment necessarily can improve the prognosis as much as possible.

## Introduction

*Ureaplasma urealyticum* (UU) is an opportunistic pathogenic microorganism, that attaches to epithelial cells and germ cells, and colonizes on the mucosal surface of the urogenital tract of adults or the respiratory tract of infants (1). It is a common pathogen in the reproductive tract of women of childbearing age, and the detectable rate of women in pregnancy can be as high as 82% (2). The UU infection causes reproductive tract inflammation, infertility and adverse pregnancy outcomes, such as premature rupture of membranes (PROM), chorioamnionitis and more (3, 4). Studies have reported that UU infection is closely associated with neonatal diseases, such as bronchopulmonary dysplasia (BPD), necrotizing enterocolitis (NEC) and retinopathy of prematurity (ROP) (5, 6), although it is controversial. Delayed detection and treatment of the infection leads to severe neonatal diseases and long-term effects, such as respiratory and nervous system sequelae (7). This study retrospectively analyzed the clinical characteristics, hemogram changes and disease outcomes of preterm infants with gestational age ≤32 weeks and weight ≤ 2000 g, with and without UU infection. We aimed to illustrate the influence of UU infection that may provide help for clinical workers.

## Methods

### Study Population

Preterm infants hospitalized in the Neonatology department of Shengjing Hospital affiliated with China Medical University between January 2019 and January 2021 were selected as the subjects. Inclusion criteria were the following: (a) gestational age ≤32 weeks, weight ≤ 2000 g, born in the obstetrics department of our hospital, and hospitalized in the neonatal department immediately after delivery; (b) UU detection completed within 48 h after admission (UU detection of respiratory tract, gastric fluid and urine secretions was performed in infants who underwent endotracheal intubation after birth; gastric fluid and urine UU were detected in infants without endotracheal intubation after birth). Exclusion criteria were the following: (a) infants who were not born in our hospital and transferred from other hospitals; (b) premature infants with incomplete UU detection within 48 h after admission; (c) cases with incomplete and unreliable information and unclear medical history; (d) corrected gestational age below 36 weeks at discharge; (e) infants who were discharged from hospital midway. The selected subjects were divided into the UU infection group and non-UU infection group, according to PCR detection results.

### Specimen Collection and Processing

Samples were detected by the clinical laboratory of our hospital with standard procedure, and UU RNA was detected by PCR. Samples included gastric fluid, lower respiratory tract secretions and urine of preterm infants after birth. The testing can be divided into RNA extraction and simultaneous amplification and testing (SAT). Firstly, for RNA extraction, 2 ml gastric fluid, or 2 ml urine, or 2 ml saline with respiratory tract secretions was added in sample storage tube, vortex blending. And then the sample storage tube was put in automatic nucleic acid extractor. Secondly, for PCR amplification, reaction tube with 40 μl system was centrifuged for seconds and put in sample tank; then opening the PCR detector and preheating, 42°C 1 min, to be repeated for 40 cycles. The result was judged according to the dt value. dt with no value or = 40 is negative; dt≤35 was positive. 35 < dt < 40 samples are recommended to be retested, and the retest result of dt < 40 is positive; otherwise, it is negative. A positive UU-PCR test of at least one sample after birth with 48h means UU infection is present (8–10). Automatic nucleic acid extractor was Mag-X. PCR detector was the Shanghai Hongshi SLAN Real-time fluorescent quantitative PCR detection system, and the kit was the Shanghai Rendu Biological *Ureaplasma urealyticum* nucleic acid detection kit (RNA constant temperature amplification).

### Data Collection

We acquired the completed demographic and clinical data of subjects from the medical records: (a) perinatal status: gestational age, weight, sex and mode of delivery, and maternal pregnancy status, including chorioamnionitis, PROM and gestational hypertension; (b) hospitalization period: cumulative length of oxygen use and mechanical ventilation, hemogram within 3 d after birth; (c) preterm complications: presence of BPD, NEC, brain injury, ROP and other diseases.

### Diagnostic Criteria of Diseases

The BDP diagnostic criteria referred to the 2018 Workshop diagnostic consensus (11). NEC diagnostic criteria was according to Bell's staging by clinical and radiological signs (12). ROP was defined by ophthalmologic screening according to current screening guidelines (13). White matter injury (WMI) and intracranial hemorrhage (ICH) were diagnosed by clinical examination and imaging such as magnetic resonance imaging (MRI). The respiratory distress syndrome diagnostic criteria referred to the requirement of supplemental oxygen, and the clinical and radiographic features. Clinical and laboratory signs of systemic infection were necessary for diagnosis of sepsis. Patent ductus arteriosus (PDA) was diagnosed by echocardiography. Cutoff values of ALP >900 IU/L and serum phosphate level <5.5 mg/dL defined metabolic bone disease in preterm infants (MBDP) (14).

### Statistical Analyses

The SPSS 23.0 statistical software was applied for statistical analysis. Chi-square tests or Fisher's exact tests were used in the analyses of categorical variables. The continuous data of normal distribution were expressed by mean ± standard deviation (x ± s), and the *t*-test was used for comparison between groups. In addition, the median (interquartile spacing) [M (P25, P75)] was used for measurement data of skewness distribution, and non-parametric test was used for comparison between groups. Multivariate logistic regression analysis was used for risk factor analysis. Statistically significant difference was defined as *P* < 0.05. Receiver operating characteristic curve (ROC curve) was completed to compare the area under the curve (AUC), and its sensitivity and specificity.

## Results

### Clinical Characteristics of Study Population

In this study, 291 preterm infants were finally selected in the Neonatology department of Shengjing Hospital affiliated to China Medical University from January 2019 to January 2021. Among which UU was detected in lower respiratory tract secretions, gastric fluid or urine of 86 preterm infants, including 38 females and 48 males, with an average gestational age of 29.1 (27.9, 30.3) weeks and an average birth weight of 1257.6 ± 248.6 g. There were 205 preterm infants with negative UU test, including 93 females and 112 males, with an average gestational age of 30.4 (29.1, 31.4) weeks and an average birth weight of 1298.3 ± 287.4 g. There were no statistically significant differences in the birth weight and gender of preterm infants between the two groups (*P* > 0.05), but the gestational age between the two groups was statistically significant (*P* < 0.001). Among the perinatal factors, the rate of vaginal delivery in the UU positive group was significantly higher, than that in the negative group (*P* < 0.001), and the chorioamnionitis, PROM and gestational hypertension between the two groups were also statistical differences (*P* < 0.001, Table 1).

**Table 1:** The demographic characteristics of UU infected group and non-infected group.

**Variables**	**UU positive**	**UU negative**	***P-*** **value**
Sex, *n* (%)	86	205	*P* = 0.854
Male	48 (55.8%)	112 (54.6%)	
Female	38 (44.2%)	93 (45.4%)	
Gestational age, weeks	29.1 (27.9, 30.3)	30.4 (29.1, 31.4)	*P* < 0.001
<28 weeks, *n* (%)	22 (25.6%)	13 (6.3%)	
28~30 weeks, *n* (%)	36 (41.9%)	78 (38.0%)	
30~32 weeks, *n* (%)	28 (32.6%)	114 (55.6%)	
Birth weight, g	1257.6 ± 248.6	1298.3 ± 287.4	*P* = 0.253
<1000 g, *n* (%)	12 (14.0%)	31 (15.1%)	
1000~1500 g, *n* (%)	61 (71.0%)	127 (62.0%)	
1500~2000 g, *n* (%)	13 (15.1%)	47 (23.0%)	
Delivery mode, n	86	205	*P* < 0.001
Vaginal delivery, *n* (%)	59 (68.6%)	34 (16.6%)	
Cesarean delivery, *n* (%)	27 (31.4%)	171 (83.4%)	
Chorioamnionitis, *n* (%)	31 (36.0%)	28 (13.7%)	*P* < 0.001
PROM, *n* (%)	48 (55.8%)	54 (26.3%)	*P* < 0.001
Gestational hypertension, *n* (%)	7 (8.1%)	104 (50.7%)	*P* < 0.001
Tracheal intubation	39 (45.3%)	112 (54.6%)	*P* > 0.05
Cumulative length of oxygen use and mechanical ventilation, *n*	86	205	
Cumulative length of oxygen use, days	50.4 (30.3, 64.5)	34.9 (18.1, 50.9)	*P* < 0.001
Cumulative length of mechanical ventilation, days	33.3 (13.3, 46.4)	19.4 (9.2, 33.5)	*P* < 0.001
Differences of the postnatal hemogram, *n*	85	202	
WBC, 10^∧^9/L	10.20 (6.81, 16.15)	7.16 (5.13, 10.00)	*P* < 0.001
PLT, 10^∧^9/L	225.00 (192.00, 276.50)	204.00 (163.50, 248.50)	*P* = 0.002
MPV, fL	8.20 (7.80, 8.75)	8.15 (7.70, 8.63)	*P* = 0.633
Differences of the postnatal hemogram, *n*	67	157	
IL-6, pg/mL	60.29 (21.99, 213.4)	49.92 (19.28, 144.6)	*P* = 0.517
PCT, ng/mL	0.318 (0.262, 0.531)	0.274 (0.213, 0.418)	*P* = 0.008

*PROM, premature rupture of membranes. WBC, white blood cells; PLT, platelet; MPV, mean platelet volume. PCT, procalcitonin*.

The cumulative duration of oxygen use [50.4 (30.3, 64.5) d] and cumulative duration of mechanical ventilation [33.3(13.3, 46.4) d] in the UU positive group were higher than those in the negative group, and the differences were statistically significant (*P* < 0.001), as shown in Table 1.

The levels of white blood cells or leukocytes [10.2(6.81, 16.15) ^*^10^∧^9/L], platelets [(234.37 ± 61.49) ^*^10^∧^9/L] and procalcitonin [0.318 (0.262, 0.531) ng/mL] in UU positive group were higher than the negative group. The differences were all statistically significant (*P* < 0.05). But there was no significant difference in interleukin-6 (IL-6) level between the two groups (*P* = 0.517, Table 1).

### Outcome of UU Infection and Non-UU Infection Preterm Infants

The results showed that the incidence of BPD, ROP and MBDP in UU positive group was higher than that in negative group, and the difference was statistically significant (*P* < 0.05). The incidence of NEC, WMI, ICH, sepsis, RDS and PDA between the two groups had no significant differences (*P* > 0.05), as shown in Table 2.

**Table 2:** Outcome of UU infection and non-infection preterm infants.

**Outcome**	**UU positive (*n* = 86)**	**UU negative (*n* = 205)**	***P*** **-value**
BPD, *n* (%)	48 (55.8%)	84 (41.0%)	*P* = 0.02
NEC, *n* (%)	10 (11.6%)	23 (11.2%)	*P* = 0.920
ROP, *n* (%)	25 (29.1%)	34 (16.6%)	*P* = 0.016
WMI, *n* (%)	29 (33.7%)	49 (23.9%)	*P* = 0.084
ICH, *n* (%)	24 (27.9%)	41 (20%)	*P* = 0.140
Sepsis, *n* (%)	57 (66.3%)	134 (65.4%)	*P* = 0.881
RDS, *n* (%)	48 (55.8%)	134 (65.4%)	*P* = 0.125
PDA, *n* (%)	26 (30.2%)	56 (27.3%)	*P* = 0.614
MBDP, *n* (%)	44 (51.2%)	79 (38.5%)	*P* = 0.047

*BPD, bronchopulmonary dysplasia; ROP, retinopathy of prematurity; MBDP, metabolic bone disease in premature infants; NEC, necrotizing enterocolitis; WMI, white matter damage; ICH, intracranial hemorrhage; RDS, respiratory distress syndrome of newborn; PDA, patent ductus arteriosus*.

### Multivariate Logistic Regression Analysis of the Intrauterine Infection

The mode of delivery, PROM, and leukocyte level after delivery were independent risk factors for UU infection, while gestational hypertension was a protective factor based on multivariate logistic regression analysis with differences in univariate analysis (Table 3). As seen in Table 3, postnatal leukocyte level can be used as a diagnostic index for UU infection. After the ROC curve is completed, AUC = 0.658(*P* < 0.001), range of 0.5 < AUC≤0.7 can be used as a diagnostic index; however, the diagnostic efficiency is poor. The Youden index was 0.279, corresponding to a critical value of 11.745^*^10^∧^9/L, when the level of white blood cell after birth was higher than 11.745^*^10^∧^9/L, where UU infection could be considered.

**Table 3:** Multivariate logistic regression analysis of UU infection.

**Variables**	**OR (95%CI) value**	***P*** **-value**
Gestational age	0.97 (0.94–1.00)	*P* = 0.095
Delivery mode	0.18 (0.09–0.35)	*P* < 0.001
Chorioamnionitis	1.38 (0.64–3.00)	*P* = 0.407
PROM	2.1 (1.03–4.26)	*P* = 0.042
Gestational hypertension	0.28 (0.11–0.73)	*P* = 0.009
WBC, 10^∧^9/L	1.08 (1.03–1.13)	*P* < 0.001
PLT, 10^∧^9/L	1.00 (1.00–1.01)	*P* = 0.079
PCT, ng/ml	1.01 (0.98–1.04)	*P* = 0.585

*WBC, white blood cells; PLT, platelet; PCT, procalcitonin; PROM, premature rupture of membranes*.

## Discussion

The opportunistic pathogen UU can be divided into two biogroups, namely *Ureaplasma urealyticum* (UUA) and *Ureaplasma parvum* (UPA). The microorganism has 14 serotypes in total, among which serotypes 1, 3, 6, and 14 belong to UPA, and the other 10 serotypes are UUA. Its pathogenicity was related to different biogroups, serotypes and host factors. Perinatal UU infection is mainly transmitted vertically from mother to fetus, including intrauterine infection and intrapartum infection. The smaller the gestational age and the longer the PROM time, the greater the possibility of vertical transmission (15). A recent animal study reported that cervical epithelial damage could promote *Ureaplasma parvum* ascending infection, intrauterine inflammation and preterm birth (16). Moreover, Romero et al. reported that intra-amniotic infections are often the result of an ascending invasion (17). This study found that preterm infants born through vaginal delivery and those with smaller gestational age were more likely to be infected with UU. The UU infected mothers were more likely to suffer from chorioamnionitis and PROM during perinatal period. In addition to the observations in our population, mothers of UU positive neonates seemed to suffer from hypertension less frequently than mothers of not infected neonates. In our hospital, maternal samples for UU infection were not routinely detected in the department of obstetrics, so we diagnosed intrauterine UU infection with positive UU-PCR test of secreta or excreta of preterm infants after birth, which was a limitation of our study.

Currently, there is no lack of reports about UU infection and complications in preterm infants. UU infection can lead to many neonatal diseases that include the respiratory system, digestive system, nervous system and retina; however, most studies talk about BPD (18). BPD is a common complication of the respiratory system in preterm infants that has a complex etiology and pathogenesis. One of the most important principle mechanism is inflammation (19). Most studies considered UU infection as a risk factor for BPD, but the diagnostic criteria used in relevant studies was an outdated diagnostic criteria. We adopted the latest diagnostic criteria for BPD (Workshop diagnosis consensus 2018), and the results found that UU infection significantly increases the incidence of BPD in preterm infants (*P* = 0.02). Studies indicate that UU can induce pro-inflammatory immune responses, but may not stimulate anti-inflammatory responses in VLBW infants, that promote cytokine imbalances pushed toward a pro-inflammatory state and cause lung injury (15), thus prolonging the oxygen inhalation time and mechanical ventilation compared to the non-UU infected group (*P* < 0.001). Oxygen therapy and mechanical ventilation are risk factors for BPD, and this disease will lead to difficulty in the oxygen attainment of infants, further lengthening the time of oxygen inhalation and mechanical ventilation, forming a vicious circle. In this study, the level of postnatal leukocytes in the UU infection group were significantly higher than that the non-infection group (*P* < 0.001). The difference of postnatal procalcitonin level between the two groups was also statistically significant. But there was no significant difference in the level of IL-6 between the two groups in this study. This may be related to the short half-life of inflammatory markers that it is affected by different blood collection times.

In addition, we also found a statistically significant difference between postnatal platelet level of preterm infants with and without UU infection (*P* = 0.002). However, no relevant previous reports were reported to support these findings. Platelets play an important role in physiological and pathological process, including coagulation, thrombosis, inflammatory reaction, and maintain the integrity of vascular endothelium cell. Studies report that platelets can be used as an indicator of infection and as a guide for diagnosis and evaluation of treatment effect. Most children with sepsis have reduced platelet level (20). This study showed no difference in mean platelet volume between the two groups (*P* = 0.633); however, the platelet level in infants with UU infection were still higher than those without infection. The mechanism may be related to platelet activation caused by inflammation, which remains to be explored and confirmed.

This study analyzed the diagnostic ability of postnatal leukocyte level on UU infection, and the resulting area under the curve AUC was = 0.658, indicating its poor diagnostic efficiency. For the changes in postnatal hemogram of the infected infants over time, and whether there is a linear correlation with UU infection, or whether the values and changes can be used as diagnostic indicator for infection still needs to be confirmed with a larger sample size.

ROP is a potentially blinding disorder seen in premature infants where there is abnormal blood vessels growth in the retina. There are few reports about UU infection and ROP at present. Ozdemir et al. reported that one of the independent risk factors for severe ROP in premature infants is UU infection, and it can increase the release of vascular endothelial growth factor, which is not conducive to the growth and development of vessels in the fundus (21). MBDP is also one of the common complications that has an increased risk among those with smaller gestational age and lower birth weight (22). Current common risk factors for MBDP include nutritional deficiencies (calcium, phosphorus, vitamin D), maternal or gestational factors, mechanical factors, endocrine changes, use of certain drugs that antagonize bone metabolism, and chronic diseases (intestinal malabsorption, renal or liver insufficiency, and collagen or metabolic diseases) (23). This study shows that UU infection also increases the risk of MBDP. However, there have been no previous studies reporting the association between UU infection and MBDP, and its mechanism remains to be explored.

Some studies presented that intrauterine UU infection can promote the development of fetal lungs. The infection could be a protective factor of neonatal respiratory distress syndrome; however there was no significant statistical difference seen in the incidence of RDS between the two groups in this study (24). Similarly, there was no statistical difference in the incidence of ICH, WMI, NEC, PDA, sepsis and other diseases between the two groups. Inflammation mediated by UU infection leads to an increase in white blood cells count and inflammatory factors such as IL-8 and IL-6 in newborn blood (25).

At present, the main clinical treatment drugs are macrolides, tetracycline and chloramphenicol. Macrolides, which mainly include erythromycin, azithromycin and clarithromycin, are the most commonly used drugs for UU infection that are widely studied. Among the drugs mentioned, some scholars reported that azithromycin can improve the clearance rate of UU in infected infants, and inhibit the pulmonary inflammatory response. Its efficacy is stronger than erythromycin, reducing the morbidity and mortality of BPD. However, there is still no standard for the initial timing, optimal dose, course of treatment, and safety of azithromycin application (26). But several azithromycin therapy for chronic lung disease of prematurity (AZTEC) studies are ongoing (27). In addition, Motomura et al. found that the use of clarithromycin could reduce adverse pregnancy and neonatal outcomes induced by *Ureaplasma parvu*m (28).

## Conclusion

In conclusion, UU infection can increase the duration of oxygen inhalation and mechanical ventilation of preterm infants under 2000 g and 32 weeks, increase level of leukocytes and platelets after birth, and increase the incidence of BPD, ROP and MBDP. However, the infection, has no effect on the incidence of NEC, ICH, WMI and other diseases in preterm infants. Furthermore, delivery mode, PROM and leukocyte level after delivery were independent risk factors for UU infection. Therefore, high-risk neonates should undergo early screening of UU through a comprehensive analysis of clinical manifestations and laboratory results. This shall aid the clinician in obtaining an immediate diagnosis and providing definitive treatment to reduce complications and poor prognosis among preterm infants. However, multiple-center studies with larger scale should be done to support our conclusion.

## Data Availability Statement

The original contributions presented in the study are included in the article/supplementary material, further inquiries can be directed to the corresponding author/s.

## Ethics Statement

The studies involving human participants were reviewed and approved by Ethics Committee of Shengjing Hospital affiliated to China Medical University (2021PS674K). Written informed consent from the participants' legal guardian/next of kin was not required to participate in this study in accordance with the national legislation and the institutional requirements.

## Author Contributions

JF conceived the review and revised the manuscript. TS performed the search, collected the data, and took charge of writing the original manuscript. Both authors contributed to the article and approved the submitted version.

## Funding

This research received a grant from the funding of Key R&D Guidance Plan Projects in Liaoning Province (2020JH1/10300001).

## Conflict of Interest

The authors declare that the research was conducted in the absence of any commercial or financial relationships that could be construed as a potential conflict of interest.

## Publisher's Note

All claims expressed in this article are solely those of the authors and do not necessarily represent those of their affiliated organizations, or those of the publisher, the editors and the reviewers. Any product that may be evaluated in this article, or claim that may be made by its manufacturer, is not guaranteed or endorsed by the publisher.
